# To what extent does Tobler's 1^st ^law of geography apply to macroecology? A case study using American palms (*Arecaceae*)

**DOI:** 10.1186/1472-6785-8-11

**Published:** 2008-05-22

**Authors:** Stine Bjorholm, Jens-Christian Svenning, Flemming Skov, Henrik Balslev

**Affiliations:** 1 Department of Biological Sciences, University of Aarhus, Build. 1540, DK-8000 Aarhus C., Denmark; 2 Department of Wildlife Ecology and Biodiversity, National Environmental Research Institute, University of Aarhus, Grenaavej 12, Kalø, DK-8410, Denmark

## Abstract

**Background:**

Tobler's first law of geography, 'Everything is related to everything else, but near things are more related than distant things' also applies to biological systems as illustrated by a general and strong occurrence of geographic distance decay in ecological community similarity. Using American palms (*Arecaceae*) as an example, we assess the extent to which Tobler's first law applies to species richness and species composition, two fundamental aspects of ecological community structure. To shed light on the mechanisms driving distance decays in community structure, we also quantify the relative contribution of geographic distance *per se *and environmental changes as drivers of spatial turnover in species richness and composition.

**Results:**

Across the Americas, similarity in species composition followed a negative exponential decay curve, while similarity in species richness exhibited a parabolic relationship with geographic distance. Within the four subregions geographic distance decays were observed in both species composition and richness, though the decays were less regular for species richness than for species composition. Similarity in species composition showed a faster, more consistent decay with distance than similarity in species richness, both across the Americas and within the subregions. At both spatial extents, geographic distance decay in species richness depended more on environmental distance than on geographic distance, while the opposite was true for species composition. The environmentally complex or geographically fragmented subregions exhibited stronger distance decays than the more homogenous subregions.

**Conclusion:**

Similarity in species composition exhibited a strong geographic distance decay, in agreement with Tobler's first law of geography. In contrast, similarity in species richness did not exhibit a consistent distance decay, especially not at distances >4000 kilometers. Therefore, the degree to which Tobler's first law of geography applies to community structure depends on which aspect hereof is considered – species composition or species richness. Environmentally complex or geographically fragmented regions exhibited the strongest distance decays. We conclude that Tobler's law may be most applicable when dispersal is a strong determinant of spatial turnover and less so when environmental control predominates.

## Background

Tobler's first law of geography, 'Everything is related to everything else, but near things are more related than distant things' [1] (see review in [3]; hereafter referred to as Tobler's law), was first applied to urban growth systems, but it also applies to biological systems as illustrated by a general occurrence of distance decays in ecological community similarity [2]. Its applicability to ecology is closely related to key theoretical issues such as what determines species diversity [4] and the distribution and abundance of species [51], as well as central to the way analyses in ecology are performed [5,65]. A negative relationship between community similarity and geographic distance is often attributed to environmental gradients [2,20]. However, the 300-years old observation that environmentally similar, but non-contiguous regions harbour distinct assemblages of vertebrates and plants (Buffon's law or 'the first principle of biogeography' [6]) suggests that other factors play a role, too. Traditional explanations have emphasized dispersal limitation due to geographic barriers [20], but spatially limited dispersal can generate distance decays in community similarity even in the absence of barriers [7,8]. A negative relationship is therefore expected between community similarity and geographic distance not only as a consequence of environmental gradients, but also due to dispersal limitation [7-9]. The latter notion is strongly contrasted by the view that 'everything is everywhere, but the environment selects' (Baas-Becking's or Beijerick's law), which suggests that dispersal limitation is unimportant [10,11]. At a global scale, this view clearly does not apply to larger organisms, as epitomized in Buffon's law. Nevertheless, it has often been argued that species distributions are largely in equilibrium with environmental conditions within continents or smaller regions [12,13]. The issue is controversial, however [14], and other authors have emphasized the role of non-environmental range constraints [16], notably dispersal limitation [8,15].

When applied to ecological communities, Tobler's law has been used to refer to community similarity in terms of species composition, but communities are characterized by many other features, e.g., species richness. Large-scale variability in species richness is often argued to largely depend on climate [21,22], but many competing explanations exist [15,21,23-30]. Therefore, it becomes relevant to ask whether Tobler's law can be extended to also cover other macroecological features such as community similarity in terms of species richness and to understand the underlying drivers as well.

**Figure 1:**
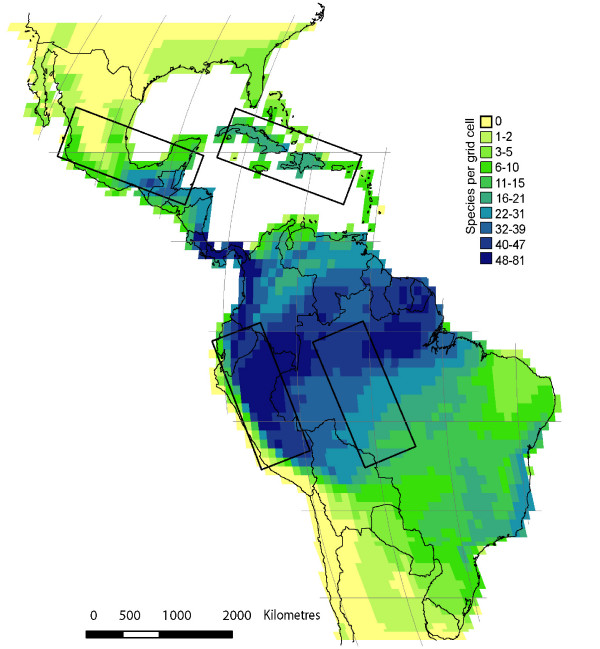
**Similarity of palm species richness and composition across the Americas**. Data compiled in 1° × 1° grid cells across the Americas. The four smaller subregions Amazon, Andes, Caribbean and Central America are marked.

Here, we use American palms to test the applicability of Tobler's law to macroecology. Palms are common in warm parts of the New World [31-33], and are particularly species-rich close to the equator [34]. Climatic water-related factors appear to be a major control of palm species richness patterns in the Americas, but nonetheless there are also historical and unexplained broad-scale spatial patterns [34,35]. Previous studies of distance decays in palm species composition have focused on local to regional scales [36,37]. In this study, we use distribution data on palm species richness and composition across the Americas to investigate the general applicability of Tobler's law to palm macroecology. Specifically, to obtain a deeper understanding of the mechanisms controlling distance decays in similarity of species composition and richness, we assess the following three key hypotheses: (1) If species composition is more strongly influenced by dispersal limitation than species richness, a stronger, more regular distance decay is expected for similarity in species composition. (2) As a further corollary, geographic distance will have a stronger impact than environmental distance on the distance decay in similarity in species composition, whereas the opposite will be true for species richness. (3) Comparing different regions within the Americas (Figure 1), the strength of the distance decay in community similarity will be positively correlated with the heterogeneity and complexity of the region, i.e., strongest in environmentally complex (*e.g*., mountainous regions) or geographically fragmented regions (*e.g*., island archipelagos). The former may reflect either the direct effect of the environmental gradients or the many barriers to dispersal in environmentally complex regions, while the latter more unambiguously reflect limited dispersal.

## Results

### Distance decay in palm species richness and composition

**Figure 2:**
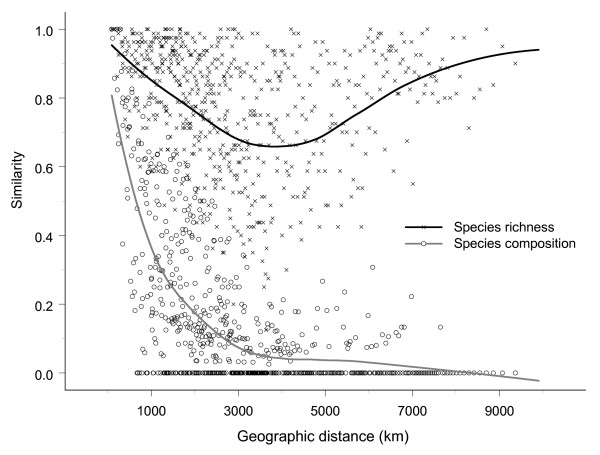
**Distribution of palm species richness**. Similarity as a function of geographic distance between 1° × 1° grid cells. Fits are quadratic Gaussian loess fits with automatic span selection (S-PLUS 7.0). Only every 2000th data point is shown.

The distance decay for palm species richness is weaker and less consistent than the decay for palm species composition across the Americas. The similarity of species richness declines over the first 4000 kilometers, but then increases again (Fig. 2), reflecting that species richness is high in the central, equatorial part of the Americas and low towards the northern and southern limits of our study area (Fig. 1). In contrast, similarity in species composition decreases approximately exponentially with geographic distance over the entire study area (Fig. 2). The decrease is very steep over the first 4000 km, where after the similarity slowly approaches zero.

**Figure 3:**
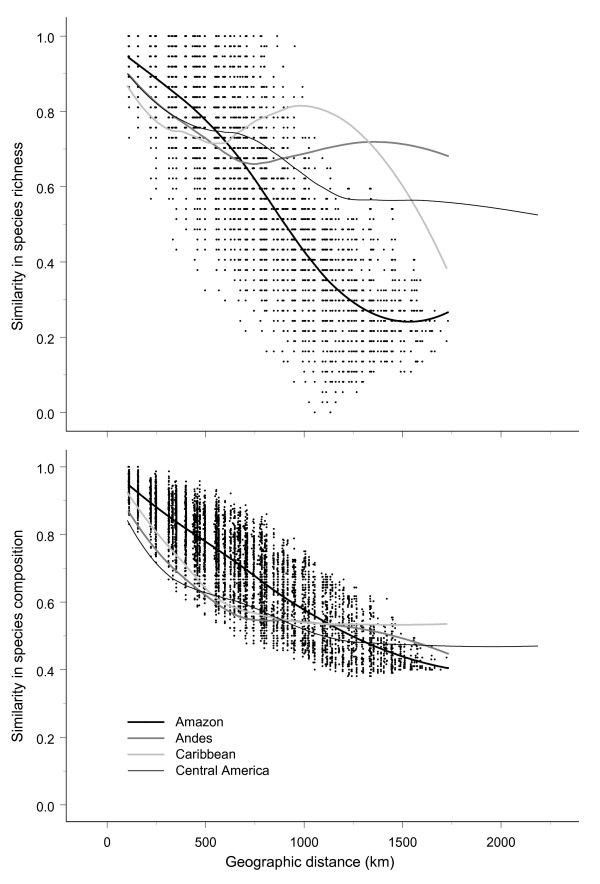
**Similarity of palm species richness and composition in the four subregions**. Similarity as a function of geographic distance between 1° × 1° grid cells. Fits are quadratic Gaussian loess fits with automatic span selection (S-PLUS 7.0). Data points are only shown for the Amazon subregion.

**Figure 4:**
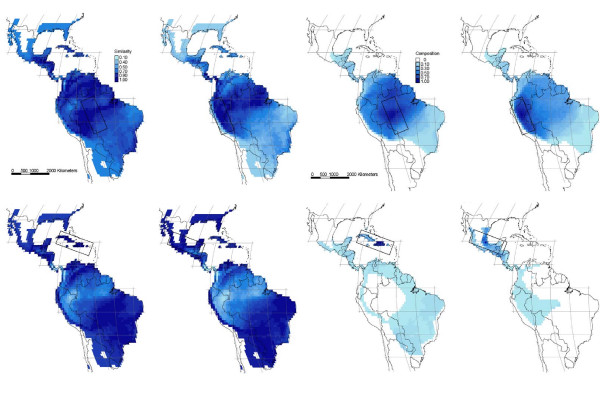
**Similarity in species richness and composition per 1° grid cell in the four subregions**. Percentage of similarity in species richness (4 maps to the left) and composition (4 maps to the right) between one single grid cell in the center of each subregion and all other grid cells within the study area. The subregions are indicated on the individual maps.

Within the four subregions (Table 1), both aspects of community similarity exhibited distance decay (Fig. 3 &4), but it was less regular for species richness than for species composition in the Andes, Caribbean and Central American subregions (Fig. 3 &4). At small distances, the distance decay was always strongest for similarity in species composition, as shown by the lower initial similarity values (Table 2). The same was true at larger distances, as indicated by lower quartile distances, with the exception of the Amazon subregion (Table 2; see also Fig. 3 &4). The geographically and environmentally least complex Amazon subregion (Table 1) had the highest initial similarity and greatest quartile distance for species composition indicating a low beta diversity and a low species turnover even at large distances (Table 2). The Amazon subregion also had the lowest initial similarity for species richness, but, in contrast, also the lowest quartile distance for this measure (Table 2), possibly reflecting greater regularity of the distance decay for similarity in species richness (Fig. 3 &4).

**Table 1:** Descriptions of the four subregions

	*Amazon*	*Andes*	*Caribbean*	*Central America*
No. grid cells	110	107	25	71
Total species	81	124	60	81
Latitude	0.5°S-15.5°S	0.5°S-15.5°S	14.5 ° S-24.5 ° S	14.5°S-24.5°S
Longitude	55.5°W-66.5°W	69.5°W-80.5°W	64.5 ° -82.5 ° W	87.5°W-105.5°
Altitude (m) max/min	1000/5	6400/9	2900/170	5390/20
MAT* (°C) max/min	27/24	27/5	26/22	27/14
MAP** (mm yr^-1^) max/min	2700/1340	3900/190	2040/1000	2900/170

* MAT, mean annual temperature (°C).

** MAP, mean annual precipitation (mm yr^-1^).

**Table 2:** Initial similarity^† ^and quartile distance^†† ^in species richness and composition

	*Initial similarity*	*R*^2^	*Quartile distance/km*
Similarity in richness			
			
The Americas¤•	1.00	0.18*	1329
Amazon	0.96	0.72*	563
Andes	0.85	0.05*	1700
Caribbean•	(0.81)	ns	(53.295)
C. America•	0.90	0.16	814
Similarity in composition			
			
The Americas¤•	0.84	0.47*	342
Amazon•	0.91	0.72*	751
Andes•	0.81	0.18*	581
Caribbean•	0.87	0.73*	523
C. America•	0.79	0.44*	644

† Initial similarity = the similarity at a distance of 150 km. †† Quartile distance = 0.75 * initial similarity.

¤ Analyses were done for grid-pairs with a geographic distance of ≤ 4.000 km.

• Geographical distance was ln-transformed.

* p < 0.01.

### Environmental and geographic distance as controls of community similarity

Which model that best described the variation of palm community similarity varied among community measures and areas (Table 3). Across the Americas and in the subregions, similarity in species richness depended more on environmental distance than on geographic distance, whereas similarity in species composition depends more on geographical distance than on environmental distance. This is clear from both the partial regression coefficients of the best regression models (Table 3) and from the variation partitioning (Table 4). There were two exceptions to this pattern: Geographic distance was more strongly related to richness similarity and explains more of its variation in the Amazon subregion (Tables 3, 4). Conversely, environmental distance had the strongest relationship to similarity in species composition and explained more of its variation in the Andes subregion (Tables 3, 4).

**Table 3:** Multiple regression analyses of species richness (r) and species composition (c)

	*β*_(*r*)_	*R*^2 ^_(*r*)_	*β*_(*c*)_	*R*^2 ^_(*c*)_
***The Americas***		0.16^D^		0.56^D^
Environmental distance	0.365		0.259	
Geographic distance	0.092		0.619	

***Amazon***		0.73^A^		0.81^C^
Environmental distance	-0.074		0.311	
Geographic distance	0.896		0.644	

***Andes***		0.30^C^		0.44^D^
Environmental distance	0.576		0.578	
Geographic distance	-0.083		0.159	

***Caribbean***		0.23^C^		0.74^D^
Environmental distance	0.526		0.088	
Geographic distance	-0.132		0.818	

***C. America***		0.31^D^		0.53^D^
Environmental distance	0.427		0.344	
Geographic distance	0.225		0.522	

The standardized regression coefficients (β) for the best models are given. Significance levels were tested using 999 permutations. (p-values are not indicated as all results were significant (p < 0.001) due to the large sample size). The distance matrix on species richness has been calculated using Euclidean distance and the distance matrix on species composition has been calculated using D = 1- Sørensen Index. Four combinations of environmental and geographical matrices have been used and the combination for each dataset giving the best model is shown here. The letters refer to:

^A) ^All environmental variables including precipitation (mm yr^-1^), number of wetdays (yr^-1^), mean annual temperature (°C), number of vegetation types, topographic range, pH, sand (%), Ca^2+^, and CEC; linear geographic distance measured in kilometres.

^C) ^Climatic related variables including precipitation (mm yr^-1^), number of wetdays (yr^-1^), and mean annual temperature (°C); linear geographic distance.

^D) ^Climatic related variables; ln-transformed geographic distance.

**Table 4:** Partial regression analyses on species richness (R) and species composition (C)

	*Richness*				*Composition*			
		
	R_PE_	R_MX_	R_PS_	R_UN_	R_PE_	R_MX_	R_PS_	R_UN_
The Americas	0.118	0.039	0.007	0.836	0.060	0.160	0.340	0.441
Amazon	0.003	0.223	0.500	0.274	0.274	0.570	0.190	0.194
Andes	0.267	0.023	0.006	0.704	0.262	0.163	0.020	0.556
Caribbean	0.224	-0.005	0.014	0.766	0.006	0.193	0.541	0.260
C. America	0.153	0.114	0.042	0.690	0.100	0.206	0.229	0.466

Per 1° grid cell. The partition results in the four fractions: mixed spatial-environmental (R_MX_), pure spatial (R_PS_), pure environmental (R_PE_), and unexplained (R_UN_). The variation partitioning was based on regressions against the combinations of distance matrices giving the best model for each data set (See table 2).

## Discussion

### Applicability of Tobler's first law of geography to macroecology

Species richness and species composition constitute two fundamental aspects of community structure [38,39]. With respect to species composition, we found a strong geographic distance decay at the bi-continental scale (Fig. 2) and though more variable, within the four smaller regions (Fig. 3 &4). Several previous studies of similarity in species composition have shown variation with geographic distance, *e.g*., for palms and other tropical plants at local to landscape-scales [37,40] and large regional scales [8,41], boreal and temperate plants at regional to continental scales [20], terrestrial and stream invertebrates at landscape-scales [42,43], parasites on vertebrate hosts at continental scales [17,18], and terrestrial microbial eukaryotes from local to continental scale [44] (for a recent meta analysis see [2]. Since species composition so consistently exhibits distance decay, this aspect of community structure clearly conforms to Tobler's law.

Large-scale geographic variation in species richness is one of the most studied topics in biogeography (*e.g*., [21,45-48], but, in contrast to species composition, little attention has been given to the possible existence and nature of geographic distance decays in species richness. To some extent, we expect patterns of species richness and species composition to co-vary. However, since it is clearly possible for species richness to remain constant despite a complete change in species composition a tight relationship is not expected. Here, we found that similarity in species richness did not decline monotonically with geographic distance at the bi-continental scale (Fig. 2). Hence, it can be argued that geographic distance decay does not really exist for species richness at the bi-continental scale, and that, consequently, this aspect of community structure does not conform to Tobler's first law of geography. A phenomenological explanation for this result is found in the well-known latitudinal diversity gradient [49], which is also conspicuous in the American palm flora [35].

The greater applicability of Tobler's law to species composition than to species richness was further confirmed by the weaker and less regular distance decays for similarity in species richness than for species composition in three of the four subregions. A potential explanation may be that dispersal is the dominant control of similarity in species composition, while environmental conditions (in ecological and/or evolutionary time [50]) provide the main control of species richness. Distant regions can contain similar environmental conditions, *e.g*., on the northern and southern hemispheres. As a consequence, there need not be any distance decay for similarity in species richness. In contrast, given a single place of origin for each species and limited subsequent dispersal, a consistent distance decay for similarity in species composition is expected. Had species composition also been primarily determined by the environment, following Baas-Becking's law, patterns similar to those for richness would have been expected, i.e., generally less consistent and weaker or even absent distance decays. We note that consistent distance decays for similarity in species composition are also expected from the phenomenological perspective that species-range size frequency distributions are generally right-skewed, i.e., most species ranges are small [51].

### Stronger distance decays in environmentally complex or geographically fragmented regions

Differences in distance decays of similarity may be caused by several environmental factors, taxa related characteristics such as dispersal properties of the species, spatial configuration, extent, and grain size [14,17,20]. These are not mutually exclusive, but likely to interact [20]. In spatially heterogeneous environments, the frequent occurrence of highly unsuitable environmental conditions (*e.g*., high mountain ridges) may act as barriers to dispersal and generate particularly strong distance decays in community composition. In geographically fragmented regions such as archipelagos, sea areas constitute strong barriers to dispersal for many terrestrial organisms, again resulting in strong distance decays in community composition. The hypothesis that the distance decay in community similarity would be strongest in environmentally complex or geographically fragmented regions was confirmed by our results (Table 2) supporting the view that dispersal can be limited by geographic barriers, and hence that community similarity is not alone 'selected by the environment' [10,11].

### The importance of environmental and geographic distance

The relative importance of dispersal limitation and environmental determination is a key issue in studies of species distributions and beta diversity [8]. A similar discussion is also a key focal point in studies of large-scale gradients in species richness although, in this case, the alternative to environmental control is considered to be historical factors in general [30,52]. Time effects (time-for-speciation, time-for-immigration) are prominent among historical explanations of species richness patterns, and clearly involve dispersal limitation at the species or above-species levels [53-55]. Nevertheless, as stated in our third study hypothesis and discussed earlier, dispersal is expected to pose a stronger constraint on species composition than on species richness, while the opposite is true with respect to environmental conditions. Our results for New World palms generally provide support for this hypothesis. Hereby, additional evidence is provided for the greater importance for dispersal as a control of species composition and a greater importance of the environment as a control of species richness.

Environmental distance was always the dominant control for similarity in species richness (Tables 3, 4), except in the Amazon region. In contrast, the relative importance of geographical and environmental distance for similarity in species composition seems to depend on scale. We found geographical distance to be a stronger control of similarity in species composition at the bi-continental scale than in the smaller regions (especially in terms of variation explained, Table 4), except in the geographically fragmented Caribbean regions, where dispersal limitation would expected to especially strong. The weak role played by geographic distance in the Andes can be expected by the close juxtaposition of highly divergent environments and strong longitudinal barriers in this region. In a previous study of palm communities in a small subregion of Amazonia, the relative importance of geographic and environmental distance was also scale-dependent, with geographic distance dominating at the regional scale, while environmental distance dominated within single localities [37]. Including somewhat larger distances, a study on palm communities in the western Amazon basin reported that geographic distance was more important than environmental distance as a control of similarity in species composition [40], while environmental distance predominated in a local-scale (50 ha) study of Amazon palm species composition [56]. Similarly, Harrison *et al. *[57] found that in 15 taxa (including plants, vertebrates and invertebrates) beta diversity was determined by the spatial structure of the environment, and argued that the influence of distance would only be important at larger distances. Our results corroborate this idea, suggesting the distance, and by inference dispersal, becomes more important as the spatial extent increases.

## Conclusion

We conclude that the applicability of Tobler's first law of geography differs among different aspects of community structure, *i.e*., it is strongly applicable to species composition and only partially applicable to species richness. It appears that Tobler's law is most applicable when dispersal limitation is a strong determinant of community structure and less applicable when environmental control predominates. Corroborating this interpretation, the applicability of Tobler's law to species composition appears to increase with increasing spatial extent, *i.e*., with increasing likelihood of dispersal limitation. As a general hypothesis, we propose that Tobler's law is highly applicable to aspects of macroecology that depend on the single place of origin of each species and the limited dispersal abilities of most macroscopic organisms. In contrast, we expect Tobler's law to be much less applicable to aspects of macroecology that are largely driven by the abiotic environment, as abiotic conditions are often similar in highly distant locations.

## Methods

### Study species

Distributional data was obtained by scanning all 550 palm species distribution maps from Henderson *et al*. [33]* Field Guide to the palms of the Americas*. These maps, the only data on palm distributions currently available for all of the Americas, were digitized and georeferenced in ArcView 9.0, ESRI Inc., Redlands, California, USA at a 1° × 1° grid square resolution.

### Study area

Our analyses were done for the entire tropical to warm-temperate parts of the Americas (34°N – 34°S; 33°W – 120°W; 1567 grid cells) and for four subregions (700 km × 1800 km, covering 110 grid cells each) in contrasting geographic and environmental settings and placed as parallel pairs at two latitudes. Grid cells with less than 25% land cover or without palm records were excluded (Table 1). The four subregions and their geographic and environmental setting were:

1. The Amazon subregion, which has a weak north-south gradient in temperature, precipitation, and topography and has not been exposed to major tectonically events for millions of years [58]. Geographically and climatically it is the least complex among the four studied subregions.

2. The Andean subregion, which includes portions of the Ecuadorian and Peruvian cordillera and its foreland stretching into the Amazonian basin (Fig. 1, Table 1). This complex region spans a broad range of temperatures and precipitation and is geologically young, resulting from a major uplift in Late Miocene about 5 million years ago [59].

3. The Caribbean subregion, which covers the Greater Antillean archipelago formed during the Eocene 55–35 millions years ago [60]. This geographically fragmented and topographically diverse island region (Fig. 1, Table 1) located just south of the Tropic of Capricorn has a more seasonal and less humid climate than the equatorial regions.

4. The Central American subregion, which covers large parts of Mexico including most of the Yucatan peninsula, Guatemala, Belize and part of Honduras (Fig. 1, Table 1). It is climatically and topographically complex.

### Environmental variables

For each grid cell nine explanatory environmentally related variables were computed: (1) mean annual temperature (°C); (2) annual precipitation (mm yr^-1^); (3) number of wet days per year (variables 1–3 were obtained from [67]); (4) topographical range (maximum – minimum elevation, extracted from the Digital Elevation Model from United States Geological Survey [68]; (5) number of vegetation types, computed from a vegetation map with a resolution of 1:20,000,000 [61] using the majority type option in the Zonal Statistics function in Spatial Analyst [62]; (6) soil pH; (7) percentage of sand; (8) soil cation exchange capacity; (9) percentages of CaCO_3 _in the soil (variables 6–9 describes 0–30 cm topsoil properties and were obtained from FAO's Digital Soil Map of the World, Version 3.5, November 1995). The variable land cover describes the percentage of land in each grid cell. The residuals from a regression between land cover and number of species per grid were used in parallel analyses. However, the influence of land cover turned out to be negligible (results not shown).

### Distance matrices

All distance matrices were computed in R-package version 4.0 d6 [63]. All environmental variables were standardized and converted into Euclidean distance matrices. For species richness analyses, we used two different environment matrices, one based on all nine environmental variables (environmental distance) and one based on three climate variables (climatic distance). For species composition analyses, topographic range and number of vegetation types were excluded from the computation of environmental distance, as species composition is not expected to be related to measures of environmental heterogeneity.

Geographic distance between grid cells was calculated as the distance in kilometers between the grid cell centroids. Two geographic distance matrices were used, one based on the linear distance and one based on the ln-transformed distance. Dispersal limitation is expected to cause logarithmic distance decay according to Hubbell's neutral model [8].

Similarity in species composition was computed using the Sørensen index, while similarity in species richness was based on the Euclidean distance (D), converted to a similarity (S) using the formula S = 1 - D/D_max_, where D_max _is the maximum distance observed. Community similarity was analyzed directly or after ln-transformation [17,19,20].

### Data analyses

To obtain an estimate of the strength of the distance decay in community similarity, we calculated initial similarity following Soininen *et al. *[2]. In our case, initial similarity was defined as the similarity at a distance of 150 km, to ensure that we did not calculate the similarity within just one 1° × 1° grid cell (approximately 110 km * 110 km close to the Equator) (Table 4). Furthermore, we calculated the distance at which the initial similarity was 75% of its original value (the quartile distance). This measure was inspired by Soininen *et al*.'s [2] halving distance, but we were not able to measure the halving distance in all subregions as the similarity sometimes did not drop below 50%. We used two different calculations depending on the form of the original regressions, linear-linear (y = α + β × x) or log-linear (y = α + β × lnx) (y = similarity at the distance x, α and β being the regression parameters) (Table 2). Initial similarity reflects turn-over of species richness or composition at relatively small spatial distances, while the quartile distance describes turn-over at broad spatial distances [2].

The importance of geographic and environmental distance as controls of community similarity was analyzed using multiple regression analyses on distance matrices [64]. Multiple regressions were run for the entire study region (the Americas) and the four subregions, separately. Four combinations of explanatory distance matrices were used: (A) environmental and linear geographic distance, (B) environmental and ln-transformed geographic distance, (C) climatic and linear geographic distance, and (D) climatic and ln-transformed geographic distance. The best model was selected as the model with the highest R^2^. The multiple regression analyses on distance matrices were done using Permute 3.4! with levels of significance assessed by a permutation procedure (999 permutations) that take into account the non-independence of the similarity values [64].

We partitioned the community similarity variation into its pure environmental distance (R_PE_), pure geographic distance (R_PG_), mixed geographic-environmental distance (R_MX_), and unexplained (R_UN_) fractions using partial regressions [14,19,36,65,66]. Variation partitioning was done for both measures of community similarity and for the entire study region as well as each subregion. For each data set, the best of the four models described above was used as the basis for the partitioning. Multiple regressions on both the environmental and the geographic distance matrices, the environmental distance matrices alone, and the geographic distance matrices alone were computed to obtain the total explained variation (R^2 ^= R_T_), the variation explained by geographic distance (R_S_), and the variation explained by environmental distance (R_E_). Based on these values, the pure geographic distance, pure environmental distance, mixed geographic-environmental distance, and the unexplained fractions of the variation in community similarity were calculated as R_PG _= R_T _- R_E_; R_PE _= R_T _- R_G_; R_MX _= R_T _- (R_E _+ R_G_) and R_UN _= 1 - R_T _[61].

## Authors' contributions

SB compiled the GIS-data with help from FS. Data analysis was performed by SB and J-CS. The manuscript was written by SB, J-CS and HB. All authors read and approved the final manuscript.
